# ‘I don't know what to do or where to go’. Experiences of accessing healthcare support from the perspectives of people living with Long Covid and healthcare professionals: A qualitative study in Bradford, UK

**DOI:** 10.1111/hex.13687

**Published:** 2022-12-13

**Authors:** Sarah A. Baz, Chao Fang, J. D. Carpentieri, Laura Sheard

**Affiliations:** ^1^ Department of Health Sciences University of York York UK; ^2^ Institute of Education University College London London UK

**Keywords:** access, ethnic minority, healthcare support, inequality, Long Covid, qualitative

## Abstract

**Background:**

In October 2022, it was estimated 2.3 million people in the United Kingdom have self‐reported Long Covid (LC). Many people have reported not receiving adequate healthcare support. There is a lack of research which provides an in‐depth exploration of the barriers faced by people with LC in accessing healthcare support. It is important to understand these barriers to provide better support, care and advice for those experiencing LC.

**Objective:**

To understand the barriers faced in accessing primary, secondary and specialist healthcare support for people with LC.

**Design and Participation:**

40 interviews were conducted with people living with LC in Bradford alongside 12 interviews with healthcare professionals (HCPs) providing LC support in Bradford healthcare settings. Interviews were analysed using reflexive thematic analysis.

**Results:**

People living with LC had a large degree of difficulty in accessing healthcare services for LC support. We categorized the healthcare access experiences of participants into five main types: (1) being unable to access primary care, (2) accessing primary care but receiving (perceived) inadequate support, (3) extreme persistence, (4) alternatives to mainstream health care and (5) positive experiences. There was a severe lack of access to specialist LC services. Ethnic minority participants faced a further barrier of mistrust and fear of services deterring them from accessing support. HCPs discussed systemic barriers to delivering services. Experiences were embedded in macrostructural issues further exacerbated by the pandemic.

**Conclusion:**

To better support people with LC, the barriers faced in accessing healthcare support must be addressed. Of significance, improvements to general practitioner access are required; especially as GPs are the first line of support for people living with LC.

**Patient and Public Involvement:**

A patient and public involvement group is engaged at regular intervals in the project.

## 1 INTRODUCTION

Long Covid (LC) is a rapidly emerging medical condition that first drew headlines nationally and internationally in 2020.^1^ In the early stages of the pandemic, many medical professionals and patients reported being neglected or disbelieved about their persisting COVID‐19 symptoms.^2^, ^3^ Thus, they mobilized online via social media to create awareness of their condition. As such, LC is believed to be the first illness constructed by patients.^2^, ^4^ Despite the increasing prevalence of LC, its definitions remain vague and are continuously evolving. Adopting the WHO definition, NICE states that the term LC ‘is commonly used to describe signs and symptoms that continue to develop after acute COVID‐19. It includes both ongoing symptomatic COVID‐19 (from 4 to 12 weeks) and post‐COVID‐19 syndrome (12 weeks or more)’.^5^ ^,p.5^ Post‐COVID‐19 syndrome is described as presenting with a cluster of often overlapping symptoms which fluctuate, change over time, affect any system in the body^5^ and impact ‘everyday functioning’.^6^ ^,p.674^ Symptoms of LC include breathlessness, fatigue, cough, fever, neurological symptoms (such as loss of taste and smell and brain fog), skin rash and chest pain.^7^ There has been an increasing emergence of academic studies exploring LC and the medical and social impacts it has on people's lives.^7^, ^8^, ^9^, ^10^, ^11^

Box 1 **Patient and Public Contribution**

1 *Designing an interview schedule for people with LC*: The wider CONVALESCENCE research project has a patient and public involvement (PPI) group involved in various work packages. The PPI group is hosted by researchers at the University of West of England who have expertise in patient and public involvement. Members of the PPI group all have or had LC. After an extensive literature review,^4^ a draft of the interview schedule was presented to the group via a workshop. Feedback suggestions included simplifying the language of questions and approaching questions sensitively. The interview schedule was then revised and piloted by the research team for further refinement (see Appendix A).

*Data interpretation workshop*: Following the advice of the UWE researchers, the PPI group were presented ahead of time with four interview transcripts from the data set and provided their interpretation of the interviews via a workshop. The theme of barriers to accessing health care was also highlighted by attendees, for example, they discussed patients being disbelieved (particularly young people) and fragmented services as some points of interest within the transcripts.

The United Kingdom has universal healthcare provision, which is free for most at the point of delivery.^12^ However, barriers to access are impacted by a healthcare system which has faced years of austerity, budget caps, increasing waiting times, pressurized services, backlogs and workforce shortages.^12^, ^13^ This has been further exacerbated by the pandemic, consequently impacting people's ability to access health care. COVID‐19 has been said to have created a ‘perfect storm’ ‘interacting with and exacerbated by social, economic and health inequalities’.^12^ ^,p.3^ The pandemic has further intensified health inequalities, and existing chronic health and social conditions.^12^ Healthcare services are fragmented, with patients transitioning between multiple care pathways; often, patients consult with GPs who act as gatekeepers to other specialist services.^14^, ^15^ Given the complexities and uncertainties surrounding the diagnosis, treatments and impacts of LC, it is expected that it may become a burden upon the healthcare system.^16^ Although some studies and commentary pieces have touched upon LC patients not being believed by healthcare professionals (HCPs) leading to them managing symptoms alone,^7^, ^8^ and the importance of relationship‐based care,^17^ there is less critical analysis of nonhospitalized people's experiences of not being able to access adequate healthcare support.^7^

Moreover, there is a lack of interpretative studies that embed ethnic minority and/or socioeconomically deprived LC patients' experiences of health care within the wider structural impact the pandemic has had on the National Health Service (NHS), health inequalities and consequently, how this shapes access to healthcare services. This paper will present findings from the Bradford sample of a national qualitative study. The aim of this paper is to understand healthcare access for people living with LC.

## 2 METHODS

### 2.1 Study design and setting

This paper is based on a qualitative interview study with 40 self‐identified nonhospitalized people who are living with LC. Participants were drawn from the Born in Bradford (BiB) cohort, and further sampling via community connections in Bradford. The BiB cohort tracks the health and well‐being of over 13,500 children, and their parents over time. The second component is three interviews overtime with 12–15 HCPs and those working in/with public health supporting people with LC in Bradford. In‐depth semistructured interviews allowed people to share the lived experiences and challenges of having LC and for HCPs to share reflections on delivering care. A PPI group is engaged at regular intervals in the project (Box 1).

Bradford is a city in the North of England with high levels of deprivation, poverty and health inequalities.^18^ As such, we engaged with a socially and ethnically diverse sample. Bradford experienced a high number of COVID‐19 cases compared to the rest of the United Kingdom. This was cited as ‘likely to be due to greater deprivation, high population density and a higher‐than‐average number of multi‐generational households’.^19^ ^,p.1160^ Furthermore, it has been found in racial disparities report that ethnic minorities have been overexposed to and underprotected against COVID‐19.^20^, ^21^ People from deprived localities are also more vulnerable to COVID‐19 infections, both groups could disproportionately experience LC.^8^, ^12^

### 2.2 Sample and data collection

#### 2.2.1 People with LC

Interviews were conducted with 40 people living with LC in Bradford. Sampling purposively, we aimed to oversample ethnic minorities and those living in medium to high deprivation, using postcode and IMD score as a proxy for deprivation status. We approached people with a range of engagement with healthcare services and considered the severity of LC (mild to severe self‐defined symptoms). BiB cohort participants were largely in their 30s and 40s. Twenty‐one participants were drawn from the BiB cohort identified via a recurrent cohort survey. From February to August 2021, survey respondents had been asked if they had COVID‐19 and how long their symptoms lasted, with four options to choose from. Reflecting the literature at the time, although there was no firm definition of LC, it was understood to be defined as having persistent symptoms for over 4 weeks.^9^ Consequently, the main exclusion criteria were having symptoms of COVID‐19 for 4 weeks or less. We sampled respondents who stated that their symptoms were either 5–12 weeks or over 12 weeks in duration. Once the list of potential 50 participants was generated by BiB, a research assistant called respondents, inviting them to take part. Twenty‐eight were interested in taking part, the remaining 22 were either unreachable or not interested. Information sheets and consent forms were then sent out. The first author arranged the interviews. Out of the 28, 7 did not participate either because they were no longer interested or there were already enough female participants in the study, prompting us to recruit more men outside the cohort. 19 people were recruited outside the cohort through community workers and snowball sampling. Another three were approached but were not interested in participating or did not reply to correspondences. Those embedded in community settings had established trust and rapport with local people, which allowed for a diverse range of respondents to be approached.^19^ Snowball sampling was used to take a more targeted recruitment approach and engage with underserved groups, for example, those whose first language is not English and men.

Participants were predominantly female, reflecting there being more mothers registered in the BiB cohort than fathers and females being cited as having a higher risk of developing LC.^22^ Participants came from 10 different postcodes dispersed across Bradford. They worked in a range of occupations, from low‐paid/low‐skilled jobs, like warehouse workers, and professional occupations like nurses. The timeframe of when participants had their initial COVID‐19 infection was broad. Four participants had COVID‐19 at the very start of the pandemic when testing was not available. The rest of the participants had a confirmed infection via a PCR or lateral flow test as and when testing was available. Participants were at different stages of their LC illness; some had recovered, and most were still experiencing symptoms. At the time of the interview, participants had LC for a range of durations, from 6 weeks to around 20 months. There was a range of persistent symptoms reported, with a loss of taste and smell, fatigue and breathlessness being common. Table 1 provides further participant demographics.

**Table 1:** Participant demographics

Sex	
Female	29
Male	11
Ethnicity	
White British	7
Pakistani or Kashmiri	25
Indian	3
Filipino	2
White Other/Eastern European	3
Age group	
18–29	3
30–39	16
40–49	18
50–59	2
60–69	1

The interviews took place between November 2021 and March 2022. All interviews were conducted remotely by the first author either over the phone or via video call. Interviews in Urdu or Mirpuri were conducted with three people. The interviews ranged from a duration of 16 min to almost 2 h (average length of 38 min). All participants gave informed consent via a verbal audio recording at the start of the interviews. Interviews were recorded digitally and transcribed by a professional transcriber with identifiable information removed. Interviews in Urdu or Mirpuri were transcribed by the first author, with data used in outputs translated over to English.

#### 2.2.2 HCPs

HCPs were recruited starting off by contacting existing HCP contacts of the last author, followed by further snowball sampling and identification of HCPs via already recruited participants. Emails/letters were sent with an information sheet and details of what was involved. Sixteen NHS HCPs and people working with/in public health running and supporting LC services in Bradford, a key criterion for recruitment, were identified. Four did not take part as they did not meet this criterion or were unresponsive. Overall, from the 12 interviewees included, there were 3 lead clinical practitioners from Bradford LC clinics, 1 occupational therapist, 2 physiotherapists, 2 GPs, 2 service managers, 1 public health official and 1 charity CEO working with local health services. Remote interviews via video call took place from December 2021 to April 2022. Again, these were recorded digitally and transcribed professionally.

### 2.3 Analysis

A reflexive thematic analysis approach was taken.^23^ Regular analysis sessions were held by the research team (all authors) to develop themes. Healthcare access arose as a striking finding during our initial analysis sessions, with most participants discussing substantive, lengthy content about their experiences of accessing—or failing to access—various elements of the healthcare system. This participant‐driven content about healthcare access continued to dominate interviews as fieldwork proceeded. After a close reading of transcripts and analytic discussion amongst the research team, we developed a coding framework which focused on healthcare access. The first author then coded the transcripts, sense‐checking with the other authors. Reflexive thematic analysis encourages the researcher to be explicit about their subjectivities, which are considered a resource rather than a threat.^23^ During the interpretation phase, the first author drew on her expertise in ethnicity and health, and the last author drew on her expertise of macrolevel healthcare systems, to situate the findings within both the Bradford and national context. The first author conducted further reflexive and interpretative work to analyse and write up the paper, producing a coherent narrative about healthcare access for LC rather than reporting basic ‘facts’ about the topic.^24^ HCPs perspectives were analysed in relation to healthcare access, integrating the two data sets. The interviews were analysed in Microsoft Word without any software package.

## 3 FINDINGS

We were immediately struck by the difficulty the majority of participants faced when accessing healthcare support for LC. This was a vociferous and very clear overarching narrative which proceeded throughout data collection. We applied the following research question to the data to help us make sense of what participants were telling us: ‘What happens when patients try to access care and support for their LC symptoms?’ We found this could be delineated into three themes. First, the differing experiences that participants had of healthcare access, which we break down into five main types. Second, experiences of mistrust and fear among ethnic minorities in our sample. Third, systematic barriers to service delivery which was an issue discussed predominantly within the HCP interviews.

### 3.1 Experiences of accessing healthcare

We found five main types of experience that participants discussed when accessing or trying to access healthcare support for LC. First, some people with LC were not able to get through to primary care and were not able to secure a general practitioner (GP) appointment. Second, many were able to access primary care but did not receive (perceived) adequate support from either their GP or secondary care. Third, a small group of participants who were extremely persistent in their interaction with health care sought LC support. Fourth, a group used alternatives to accessing mainstream health care for various reasons. Fifth, a small number of people had positive experiences. We also discovered a severe lack of access to specialist LC clinics.

#### 3.1.1 Not getting through to primary care

Most notably, some participants were falling at the first hurdle when trying to access support and advice for LC symptoms from GP practices, often the first point of contact for patients. A common barrier was not being able to get through to practices via the phone, often facing a prolonged wait for someone to pick up, as this extract illustrates:

I will ring them and then I'm waiting on my break for like 10 minutes and nobody is answering, so I'll wait another 20 minutes. When I'm at home and I've got a day off, I don't know where to start. So I don't want to ring my doctor waiting, you know, 2 hours on the phone because I've got no time for it and I'm trying to manage my symptoms with ginger or garlic. (Interviewee 11, 40–49, female, Eastern European)

One participant was already aware that her GP was ‘extraordinarily difficult’ to get through to and instead went to her local pharmacist for advice:

I spoke to the chemist because our GP is extraordinarily difficult to get through and it's very difficult to talk to anybody other than the receptionist. So I thought I'd just go and talk to our local pharmacist and see if they can suggest anything and they just said that I've just got to let the symptoms come out naturally or take paracetamol for my headaches … relax…. there was nothing else offered if they could offer anything else I don't know…. (Interviewee 29, 30–39, female, White British)

HCP interviewees also acknowledged prolonged waiting time to get through to services as a key barrier. Resultantly, people could end up self‐managing, potentially risking further health complications:

I think there are going to be a lot of people who we're not touching. I mean it's how do you get hold of your GP? Last time it took 50 phone calls, 50 tries on my mobile. How do you do that if you're exhausted? (HCP1, physiotherapist)

A further barrier for this group was having to justify their need for an appointment with the receptionist, often facing pushback. For example, when interviewee 34, a British Pakistani male in his early 30s, contacted his GP, he felt that he was not a priority. He mentioned the LC clinic to the receptionist, however, had to face a long waiting time of 3 weeks for an initial appointment with the GP before referral, leaving him to state: ‘So in my mind at that time it was just kind of that natural response to when you're being pushed back to say, “okay I'll leave it then” and that was that’. Thus, being able to get a GPs appointment in the first instance is one major barrier many people with LC are facing. But those who were able to eventually get through also faced hurdles within the healthcare system.

#### 3.1.2 Accessing primary care but receiving (perceived) inadequate support

Most participants were able to get through to their GP and received an appointment but felt they had received (perceived) inadequate support from primary care. An interview with a couple with LC, living in a deprived area of Bradford, provides one account of such experiences. The difficulty of being able to access their local GP was further exacerbated during the pandemic. When they finally got through, they were not happy with the advice given:

Wife: …They said just take paracetamol.

Husband: This is normal. This is common in here. Take paracetamol and ring two months. (Couple interview, 40–49, Pakistani)

Some participants described a sense of disappointment in primary care. One participant stated that he felt ‘hopeless’ and ‘neglected’ (Couple interview, 40–49, male, Pakistani). Participants wanted to receive more advice and support from their GPs. Another participant felt that LC was not taken seriously compared to other medical conditions, a common finding reported in previous studies^8^, ^25^:

…when I spoke to the doctor's about feeling rubbish ‘oh well it will be just Long Covid but we don't do anything about it’. If I'd said ‘oh it's anaemia’, then they do all these tests and you can progress. But if it's Long Covid it's just ‘well that's what [it] is’. (Interviewee 20, 40–49, female, White British)

Two participants stated that they had been referred to secondary care services by their GP but were still on a waiting list after many months. Interviewee 38 had been referred to E.N.T. and was on the neurology service waiting list for 6 months but still had no answer regarding why she was experiencing persistent head and ear pains for over a year. From the interview, there was a clear sense of frustration about how long it was taking to get an appointment and navigate a fragmented healthcare service. She wanted to find answers about the cause of symptoms experienced since her COVID‐19 inflexion and had not been diagnosed with LC. The participant contemplated a private healthcare check‐up as an alternative when visiting India to see family. There she felt she would be able to get all the tests needed in one hospital visit and find some answers:

GP is also waiting for investigations and they're just giving the medications but at the end of the day I mean I'm anxious, I don't know what's happening … Wherever I go, whatever they do they're saying everything is fine. My chest x‐ray is fine … So nobody knows. They've not diagnosed it … Maybe I will go … back home in India … if I get a chance when I go back that maybe I'll go for proper treatment…. So think how many months I'm just waiting for this, you know, they could have done that CT head[scan] when I was actually there. They said no, E.N.T is only doing certain parts … I don't know what to do or where to go, to whom to ask and nothing is easy access. It takes forever … I'm really fed up with this … it's really hopeless. I'm trying to live with it now. (Interviewee 38, 40–49, female, Indian)

#### 3.1.3 Extreme persistence

We found that a high level of persistence and familiarity with approaches to get through to the right person was required to gain access to primary care. A few participants were persistent in navigating their way through services to gain medical support. These were those working in professional occupations, for example, public health, or those who had extensive previous experiences of navigating GP services because of other long‐term illnesses. Thus, they had high health literacy and access to resources. They too acknowledged the difficulties of getting through to GPs over the phone and illustrated the importance of making sure that people get through to a GP who knows their medical history and that they access continued care over time from the same practitioner,^17^ as this extract shows:

First of all they put you on a triage list and get someone to call you back and I've had to insist and say, I need to be put on my doctor's list for her to ring me. There's no point in anybody else ringing me because they don't know me. I think that's the bugbear isn't it … sometimes I've had to speak to other doctors, but they've not really known and you get mixed messaging … I just need to speak to my own because it's having that trust in somebody as well isn't it. But on the whole, I don't have any quibble … they've genuinely been supportive. (Interviewee 10, 40–49, female, White British)

Furthermore, interviewee 20, who has rheumatoid arthritis, discussed the resources that she drew upon to access primary care and be listened to:

…I think I probably I've got a bit more access than most people because of my rheumatology team. They do listen you see and because of my medication, you know, they have to listen to me. Whereas if I hadn't have had that communication and opened to me, I'm not sure it would have continued. (Interviewee 20, 40–49, female, White British)

#### 3.1.4 Positive experiences of healthcare

A few participants described having an overall positive experience of engaging with primary care for LC support. This included GPs listening, providing reassurance, practical and emotional support, receiving continuous care and follow‐up phone calls:

What I did appreciate was that my telephone call with the GP was probably slightly extended to the other ones that I have had in the past and the fact that it was the same person that I spoke to … There's an element of continuity of care that really helps. (Interviewee 1, mid‐30s, female, Pakistani)

A participant who was initially hospitalized for COVID‐19 described receiving follow‐up support from her GP, who provided practical advice on breathing exercises to help with continued experiences of breathlessness:

For my breathing, I spoke to my GP and he recommended me to like get balloons and kind of blow into them. Breathing exercises. So I used to do breathing exercises…. (Interviewee 5, 18–29, female, Pakistani)

Overall, such approaches were cited as being helpful in managing the illness and can be learnt from to provide better support to people with LC.

#### 3.1.5 Using alternatives to accessing mainstream healthcare

Every participant described a degree of self‐management or ‘burden of illness’,^26^ for example, prioritizing rest, reducing physical activity or using home remedies. However, some participants self‐managed symptoms from the offset and chose not to engage with healthcare services. This was due to several reasons, including, not preferring to approach healthcare services unless necessary, not liking medicine, preferring self‐management, not wanting to burden an already overwhelmed NHS, not knowing what help was available, mistrust, fear and past negative experiences which deterred healthcare access (see Section 3.2) and learning to live with symptoms with the hope that they would see change over time. One HCP stated that some may be ‘accepting that this is how life is for them’ (HCP5, GP).

#### 3.1.6 Limited access to LC clinics

A startling finding was that only 1 out of 40 interviewees in Bradford had engaged with an LC clinic service. This one person was an NHS staff member and had accessed an LC clinic through their workplace that was designed to help NHS staff recover and progress back to work. A very small proportion was beginning to discuss the possibility of a referral with their GP if symptoms worsened, many had not even heard of an LC clinic.

Patients have to go through a prolonged process with their GP to gain a referral to the LC clinic. A clinician interviewee stated that to gain access to the clinic, symptoms must last for 12 weeks or more. Patients must go through an initial assessment with their GP to eliminate other health risks. It was argued that this timeframe can be reduced so people can receive earlier interventions:

I think probably identifying the right people, you know, so making sure that people don't miss out. I think probably not being necessarily so strict about this 12‐week cut off, you know, because even at the moment the GPs are not allowed to refer to the community team until it's 12 weeks. But why not refer at 7 weeks, you know? Why wait? (HCP2, Physician)

Evidently, participants had to do the ‘hard and heavy work’^8^ to receive healthcare support for their symptoms. This depicts the different barriers and inequalities in accessing services among participants. The next section will further focus on mistrust and fear as an extra layer of barriers to accessing services for ethnic minorities.

### 3.2 Mistrust and fear

Mistrust and fear were pertinent issues amongst some ethnic minority participants. This has previously been cited as a barrier in relation to COVID‐19 vaccine uptake amongst ethnic minorities^19^ and creates an additional bottleneck for people with LC. A few participants expressed fear of going to the hospital for treatment of their LC symptoms. In one participant's case, this reflected ‘fake news’ stories and rumours going around the Pakistani community in Bradford at the height of the pandemic that hospitalization could lead to death.^19^ Interviewee 4 emphasized a lack of trust in doctors and a need to increase trust in healthcare services to tackle such rumours. This can be embedded in both experiences of historical and contemporary structural racism, which leads to mistrust in the healthcare system and may be further exacerbated in the case of COVID‐19, as there has been a disproportionate number of deaths amongst ethnic minority people.^27^

…you should have that full trust in him [GP] … this ‘negativity’ that is spread this this this should not happen because I understand because I I didn't go to the hospital I didn't go because of this that I heard that that's it if you go to the hospital then a person does not come back alive…. (translated from Urdu) (Interviewee 4, 30–39, male, Pakistani)

The participant stated that he attained medical advice informally from a GP, which a family member put him in touch with. The GP spoke his preferred language, and provided reassurance, advice and ‘emotional support’.

Other participants also expressed fear of being hospitalized, put on a ventilator and dying. This also reflects some participants knowing of people who have died from COVID‐19, making it more ‘real’.^19^ This was deterring interviewee 36 from seeking medical support when experiencing frightening pains in his chest due to his LC. He was yet to take the first step to engage with services:

I felt so wheezy and I felt like my chest was tightening up around me and I was really close a few times to making the call to 911[111] to say, you know, this is happening, what should I do and I couldn't do it because I was too scared to make the call.

Interviewer: ‘In what ways were you scared?’

I think like I try not to listen to people but I heard a lot of stories at the time that people were going to the hospital and not coming back out and were put on ventilators and stuff. That essentially was really scaring me and I did have one of my close friends, his brother passed away … Now that didn't scare me but it kind of puts that thing in your mind. He wasn't vaccinated. I'm vaccinated. But yeah just things like that really. I don't want to put myself in that position. (Interviewee 36, 30–39, male, Pakistani)

There were also accounts of participants lacking confidence and trust in HCPs. This was embedded in previous encounters with GP surgeries where they were misbelieved or not taken seriously. These past encounters played a part in deterring them from seeking medical advice for LC. These experiences occurred at the intersection of aged, gendered and racialized discrimination. For example, a young Pakistani woman described not being taken seriously by her GP:

I mean my doctors aren't really that good in that sense anyway, so I wouldn't even go to them for help … I went to the doctors once because I had a lump on my breast and he told me to lose weight. They didn't even check. In that kind of sense I don't go to the GP anymore because of them not being really practical about anything … it stops me…. (Interviewee 30, 18–29, female, Pakistani)

Another Pakistani man in his early 30s described feeling that as a ‘youngish man’ he was not prioritized or taken seriously and was previously ‘denied’ being given antibiotics for a medical condition, with his practice stating: ‘you're a young fit guy, you'll be fine’. Therefore, mistrust ran in both directions as patients have been mistrusted by HCPs which consequently shaped their mistrust of the system.

Often in relation to ethnic minority experiences of healthcare access, language is cited as the key barrier. However, the three Urdu/Mirpuri‐speaking participants in this study stated that their families, husband or support networks supported them in seeking medical advice. This raises the importance of shifting the conversation beyond a narrow focus on solely language to other barriers, namely mistrust and fear. This creates an additional barrier to access for ethnic minority people despite their language abilities. Past encounters of being disbelieved, having mistrust and fear can lead to a lack of confidence that adequate healthcare support will be provided, consequently impacting people's decisions on seeking support for LC symptoms, resulting in people self‐managing symptoms.

### 3.3 Systemic barriers to service delivery and access: HCPs perspectives

HCPs in Bradford shared their perceptions about the barriers people with LC face when accessing health care. There was a mix of both praise and criticism of services. However, a salient finding was the systemic healthcare access issues that HCPs had to work around.

First, there was a lack of training for GPs about LC, particularly during the onset of the illness. HCPs were overstretched and often had to figure out themselves what LC was and how to support patients, drawing on knowledge of other illnesses like chronic fatigue syndrome, and in one GPs case via her own experience of LC:

…there was nothing to offer so we were kind of winging it … making sure we weren't missing our you know bread and butter stuff erm but it just kind of felt like there was something happening to these patients that we didn't know what was happening … it was something I was reading a lot about…. (HCP12, GP)

As discussed in Section 3.1, there was limited access to specialist LC services, with an emphasis being placed on access for NHS staff with LC and patients who had been hospitalized. A physiotherapist was informed to use existing services in her own practice to support people with LC, despite already being overstretched and with increasing workloads. Nevertheless, she continued to support her LC patients:

we do more than we should and erm we work more and more and later and later and then we cannot fill all our workload … we all know that in reality it means services are overstretched ones that are already overstretched…. (HCP1, physiotherapist)

At the time of fieldwork, a newly set up LC clinic aimed to provide holistic care to Bradford patients taking a multidisciplinary approach, which is particularly key as LC is often seen as a primary respiratory phenomenon. The main barrier to accessing this clinic was the long waiting list. HCP10 (a lead clinician) stated that the clinic was ‘lagging behind’ due to the time it had taken to set up and allocate funding. Additionally, there has been a struggle to recruit staff due to shortages of specialist staff, a wider system issue which is also impacting this service.^13^

Although HCPs felt that GPs were best equipped to support LC patients, as they had knowledge of the whole body, a CEO of a third‐sector organization working with health services raised the concern of accessing the clinic via GPs, the primary route of referral. This can create an immediate ‘bottleneck’ as many face barriers to accessing GP appointments, particularly marginalized groups. This further illustrates the high importance of improving access to primary care but also using other methods to signpost patients to specialist services, including more engagement in grassroots community settings that are connected to the most underserved:

if there was a way for a wide range of groups to be able to refer, connect, signpost people to that service without having to jump through hoops for a GP then I think that will be more effective. (HCP8, charity CEO)

One GP working in a deprived locality stated that some of her patients were now able to access the clinic and shared positive experiences. However, she was yet to receive any information from the clinic on the progress made and instead had to ask patients. This reflects a fragmented healthcare system where patient record systems are not linked together between services, creating barriers to better‐supporting patients.^28^

Importantly, these findings illustrate the impact of systemic issues on service delivery and the access and support people with LC get from HCPs.

## 4 DISCUSSION

### 4.1 Existing literature

This study presents similar broader findings to existing studies into LC, particularly the experiences of being disbelieved, trying to find answers, barriers getting through to GPs, having to navigate fragmented services and self‐managing symptoms.^7^, ^8^, ^11^ However, previous findings are largely considered in the context of broader descriptive findings of the multifaceted impacts LC has on people's lives and there has been a lack of in‐depth critical exploration of the barriers to accessing health care, particularly for disadvantaged groups with LC. Previous studies have not fully captured the voices of ethnic minorities, with participants predominantly being White British.^7^ This study particularly addresses this gap, with the sample being 75% ethnic minority and mostly living in deprived areas of Bradford, which allowed us to understand how the experience of healthcare access is shaped for this group of people and capture insights about mistrust. We found five different types of experience when accessing health care alongside a lack of access to LC specialist services. Overall, it is evident that people faced worrying difficulties in accessing the healthcare system at all, with a high degree of persistence required just to access primary care.^29^ As found in previous studies, there were some positive experiences of primary care, such as GPs following up and listening,^11^ but many participants felt that their symptoms were not taken seriously.^28^ People who were referred to secondary care had to wait many months to access services. Only 1/40 of the interviewees had accessed a multidisciplinary LC specialist service, with a few people discussing the possibilities of future referrals with GPs.

It is important to embed these experiences in literature from an inequality and structural lens, given COVID‐19 and LC being both a health and socioeconomic crisis (often termed a ‘syndemic pandemic’) and experiences of access being shaped by inequalities and structural factors.^12^, ^21^ As previously argued,^7^ the sociological theory of candidacy, which describes how eligibility for care is jointly negotiated between individuals and health services, is useful here in contextualizing experiences.^29^ It is acknowledged that access requires considerable work by users and is argued that a number of factors, such as those at the material, structural, cultural, professional and individual levels, can shape the views of the most disadvantaged as to whether they are eligible for care.^29^, ^30^ Access to health care is lower in disadvantaged and deprived communities, with the number of patients per GP higher in the most deprived areas than in the least‐deprived.^12^ This results in reduced access to health care, further creating a bottleneck for people with LC. This can further contribute to inequalities and lead to worse health outcomes from LC for the most disadvantaged.^12^ Similar to previous studies on LC, participants in this study were having to still do the ‘hard and heavy’ work of both understanding and managing a new illness and navigating fragmented healthcare services.^8^, ^11^ Moreso systemic barriers, including backlogs, a decimated and underfunded healthcare system and workforce shortages, mean people with LC experience barriers to access.^7^, ^15^, ^31^, ^32^

Furthermore, this research adds further to emerging literature surrounding COVID‐19, ethnicity and mistrust amongst ethnic minorities.^16^, ^19^, ^33^ In relation to accessing LC support, experiences of mistrust and fear were rooted in the disproportionate impact of COVID‐19 on ethnic minorities, intersectional accounts of discrimination and previous negative encounters with the healthcare system.^32^, ^34^ Shahid and Dogra^32^ conceptualize this as ‘medical mistrust’. This results in fear and reduced trust in HCPs and disparities and inequalities in the utilization of healthcare services.^32^ This creates an additional barrier to accessing healthcare support for LC amongst ethnic minorities.

### 4.2 Implications for practice

Evidently, GPs are often the first point of contact for patients and play a crucial role as gatekeepers in facilitating access to secondary care and LC clinics and assessing patients.^15^, ^25^ Therefore, it is essential to improve access to primary care so people with LC are provided with better support and referral. Our study shows that this is a major barrier for LC sufferers, this was emphasized by both people with LC and HCPs. A backdrop of mistrust exists, this must also be addressed when looking at access to and engagement with healthcare services, particularly as Bradford has a diverse ethnic minority population and socioeconomic inequalities, which have led to greater risks of contracting COVID‐19. Although progress has been made in setting up an LC clinic, HCPs cited the structural barriers in the healthcare system which impacted their ability to provide support to LC sufferers. As previously cited,^14^, ^28^, ^35^ better communication between fragmented services is required so GPs can provide better follow‐up support, alongside more training and education for HCPs about LC. Wider systemic issues routed in years of austerity are evidently also impacting access and service delivery. There is concern that not everyone is able to seek help in an overwhelmed system.

### 4.3 Strengths

Earlier studies into LC focused predominately on White British populations, and HCPs with LC, and recruited participants from online platforms.^3^, ^7^, ^25^, ^36^ A key strength of this study is that it accounts for the experiences of ethnic minorities, underresearched populations (such as those with English as a second language) and people living in deprived areas, allowing us to capture their experiences of healthcare access. Another key strength of this study is that it explores HCPs' perspective of LC service delivery and access, addressing a significant gap in the literature. Future research into LC needs to explore the perspectives of HCPs in different UK settings.

### 4.4 Limitations

We do not have ‘evidence’ of COVID‐19 infection for some participants, particularly those who were infected during Spring 2020 when testing was largely unavailable. The severity of LC was also self‐defined by participants. These could be viewed as limitations, but, rather, we see the self‐identification of our sample as a positive move echoing Alwan's^37^ ^,p.201^ assertion that ‘the burden of proof should not be on ill people’. Furthermore, the data reported in this study only focuses on one city and one time point. There is a lack of longitudinal follow‐up research involving people with LC, and exploring their experiences over time.

### 4.5 Further research

Our study is the first sweep of data collection of a three‐sweep longitudinal interview study, where we will follow participants both in Bradford and across the United Kingdom over three time points over an 18‐month period. As this paper only presents the Bradford sample, we do not situate our current data as longitudinal or nationally representative. However, it is worth noting that in future publications, we will explore varying topics of importance to participants (such as LC, identity and existential loss), changes over time and whether participants have engaged with and accessed further healthcare support.

## 5 CONCLUSION

This paper has contributed to providing a more nuanced and in‐depth understanding of the barriers and ‘hard and heavy work’^8^ people with LC face in accessing healthcare support, drawing on the perspectives of people living with LC and HCPs. These subjective experiences are embedded within deep‐seated structural and systemic barriers, discrimination and health inequalities which create healthcare access barriers for people with LC.

## AUTHOR CONTRIBUTIONS

J. D. Carpentieri and Laura Sheard designed the wider qualitative longitudinal study and obtained funding as part of the CONVALESCENCE grant. Sarah A. Baz collected all the data discussed in this paper and was the primary analyst for the healthcare access focus. All authors fed into analytic discussions. Sarah A. Baz and Laura Sheard wrote the first drafts of the paper. All authors read, commented and provided feedback on the paper, providing approval for the final manuscript.

## CONFLICT OF INTEREST

The authors declare no conflict of interest.

## ETHICS STATEMENT

This study has been ethically reviewed and given a favourable opinion by the University of York Health Sciences Research Governance Committee on 01/10/21 (ref: HSRGC/2021/466/B).

## Data Availability

The data that support the findings of this study are available on request from the corresponding author. The data are not publicly available due to privacy or ethical restrictions.

## APPENDIX A INTERVIEW SCHEDULE

1. **Introduction**
  We are interested in talking to you about your experience of living with symptoms of Covid. We know from the Born in Bradford survey you stated you had Covid symptoms for more than 4/5/12 weeks. Thanks very much for answering questions about Covid on the survey. Today, I'd like to have a more in‐depth conversation about your experiences of living with Covid.
  We are aware that some people experience longer Covid symptoms than others. You may have heard of the phrase ‘Long Covid’ as it is an increasingly popular term (although we are aware that not everyone with Covid symptoms for more than a month would identify as having ‘Long Covid’). We are carrying out this study to understand more about the experiences of living with Covid symptoms for about 5–12 weeks or more. We aim to provide evidence to improve practice and policy.
  This interview will explore the impact that Covid has on your everyday life and your experiences of accessing healthcare support for Covid symptoms. The interview will last between no more than 1 h. Importantly, you do not have to answer any questions you are not comfortable with. You can also stop or pause the interview at any time. You have the right to withdraw during and after the interview—any data collected will be destroyed if you decide to withdraw. If you would like me to repeat any question or provide further explanation, please feel free to ask. You can also ask questions at any time during the interview.
2. **Opening question/ice breaker**
  (i) Tell me a bit about yourself (e.g., are you working/studying/retired)
  2.1. Initial experience of Covid
    (I) Can you remember when you first had COVID‐19 symptoms?
    (II) Tell me about your initial COVID‐19 experiences.
    (III) Did you get a test? (lateral flow or PCR?) What happened?
    (IV) Did you have any contact with your GP, the hospital or other healthcare services? Can you tell me more about that?
    (V) What support did you seek when you got Covid‐19?
  2.2. Thoughts about Long Covid and experiences of prolonged symptoms
    (I) What do you think about the term ‘Long Covid’ and do you think it applies to you? If not, why not and what would be a better term?
      (From this point onwards, use the patient's self‐defined term to describe their Covid symptoms)
    (II) Do you experience any Covid related symptoms now?
    (III) How long do you think your Covid symptoms have lasted?
    (IV) Would you say your health was good before getting Covid? If not, what conditions did you have?
3. **Managing the illness**
  3.1. Impact on day‐to‐day life
    (I) How have the Covid symptoms affected your life?
    (II) What changes have you noticed—describe how your daily routine has changed compared to your days before having Covid (e.g., could you give me an example of a typical (week)day before getting Covid and then a typical day after getting it?) (Dyad question)
    (III) How did you try to maintain your normal routine? Did you find it was challenging?
  3.2. Managing symptoms
    (I) Tell me about the symptoms you have experienced over time. Did the Covid symptoms change?
    (II) What sort of things have you been doing to help manage/cope with your symptoms?
    (III) Have you got any strategies to manage the symptoms? If yes, what are the strategies?
    (IV) How have your physical activities been affected by your Covid symptoms? What changes have you noticed? (e.g., is there an activity you are no longer able to do/you find more challenging now compared to pre‐Covid?)
  3.3. Impact on mental health
    (I) Has your Covid experience impacted your well‐being in any way? (feeling frustrated, stressed, sad, angry)
    (II) How are you feeling now regarding your experience of having to live and cope with Covid?
4. **Healthcare services**
  4.1. Experiences with GPs/hospitals/health services
    (I) Have you asked for medical advice, support or treatment for Long Covid?
    (II) Did you contact your GP? What happened? Who did they refer you to?
    (III) What advice were you given and was it useful?
  4.2. Barriers and levers to access
    (I) Have you faced any barriers or difficulties when accessing health care? If so, what are they?
    (II) Can you tell me about anything that was good about accessing healthcare support that helped you.
  4.3. Support required/interventions
    (I) What further healthcare support do you think would help with your recovery?
    (II) Reflecting on your experiences, what improvements do you think are needed to better support people with similar experiences as you? Why do you think so?
  4.4. Vaccination (optional)—If participants do not wish to discuss this, we will skip the questions below.
    (I) If you don't mind, can you tell me if you have received the Covid vaccine? If so, how many doses have you received?
    (II) Do you think vaccines have helped you recover from your symptoms? If so, how?
5. **Role of family and friends**
  5.1. Impact on family life/relationships
    (I) Tell me a bit about your family or those living with you.
    (II) Did you seek and/or receive support from your family or friends? If so, what support?
    (III) How has your illness impacted them and your relationship with them (This may not be asked in a dyad interview)
  5.2. Responsibilities
    (I) How have your Covid symptoms impacted your ability to (e.g., participate in family life, volunteer, work)?
    (II) What changes or difficulties have you and your family members experienced? (e.g., increased caregiving burden, changing family roles).
    (III) Do you have any caring responsibilities in your family/social circle? (e.g., childcare, caring for an ageing/sick family member/friend).
    (IV) How have your Covid symptoms affected your ability to provide care?
    (V) How have the changes in caring responsibilities altered your relationships/roles in your family? How did you cope with these changes/difficulties?
      For dyad family members/friends
    (VI) How important has the support from your partner/family member/friend been while having Long Covid? What aspects have you both struggled with?
    (VII) How did your family/friend's Covid experiences impact your life and/or the whole household?
6. **Online support groups**
  (I) Have you accessed support from any Long Covid support groups, either face‐to‐face or online (e.g., Twitter, Facebook group)?
  (II) What is your experience of engaging with these groups?
  (III) How has this shaped your experience of living with Long Covid?
7. **Socioeconomic impact**
  7.1. Impact on employment
    (I) Do you work? (Yes—What job do you do? No—Did you work before having Covid?)
    (II) How has Long Covid impacted your ability to work?
    (III) Do you think your employer was helpful in terms of supporting you during your Covid illness? If so/or not so, could you expand your answer further?
    (IV) Have you experienced any barriers when returning back to your normal work routine? If any, can you further explain your answer to me?
    (V) What support did/do you require to get back into work?
  7.2. Financial impact and benefits (prompt answering is optional)
    (I) Have you experienced changes in your finances after getting Covid? (and reducing work hours/losing job/receiving benefits)
    (II) What are your experiences of leaving/reducing employment due to Covid? What support did you receive from your family, communities and/or the Government?
    (III) There have been discussions about including patients with prolonged Covid experiences into the disability benefits (e.g., using this as an eligibility criterion for certain types of benefits). What do you think?
      For dyad family members/friends
    (IV) How has the impact of Covid on your partner's employment/finances impacted the household?
8. **Impact on identity**
  (I) Has Long Covid changed who you are? (identity as parent or worker, etc.)
  (II) Thinking about your future: First, what are your hopes, in terms of Covid and your health, and any of the impacts you've talked about today?
  (III) Do you have any fears? What are they?
  (IV) If you could receive more support in the future regarding your Covid experiences, what kind of support would you like to receive and from where/whom?
  (V) (For recovered participants) What support would you like ongoing Long Covid sufferers to receive?
    For dyad family members/friends
  (VI) Do you think Long Covid has changed your family/friend in terms of (e.g., used to be active and sporty, someone with confidence, breadwinner for the family)?
9. **Finishing off**
  (I) Is there anything else you would like to add? Anything we haven't covered or you want to discuss more about?
  (II) Do you have any questions?
10. Debrief: Thank you for taking part in this interview. Please feel free to email or phone me if you have any further questions. Over the course of the project, we will inform you about outputs from the project. I will also be in touch around May 2022 to arrange the second interview—I will be in contact closer to the time.
